# Meaning-centred intervention for managing loneliness among community-dwelling older adults: a mixed-methods systematic review protocol

**DOI:** 10.1186/s13643-025-02843-x

**Published:** 2025-05-08

**Authors:** Ken Hok Man Ho, Jackie Hoi Man Chan, Daphne Sze Ki Cheung, Wallace Chi Ho Chan, Paul McQuillan, Mei Tim Yu, Malik Gulzar, Cho Lee Wong, Chaojie Liu

**Affiliations:** 1 School of Nursing and Midwifery, La Trobe University, Melbourne, VIC 3086 Australia; 2 School of Humanities, Social Sciences and Health, University of Wollongong Dubai, Dubai, United Arab Emirates; 3 School of Nursing and Midwifery, Deakin University, Melbourne, Australia; 4 Centre for Quality and Patient Safety Research/Alfred Health Partnership, Institute for Health Transformation, Deakin University, Melbourne, Australia; 5 Department of Social Work, Education and Community Wellbeing, Northumbria University, Newcastle, NE1 8 UK; 6 Lifechange Therapies, Oxley, Brisbane, QLD 4075 Australia; 7 Chinese Health Foundation of Australia, Melbourne, Australia; 8 Nethersole School of Nursing, The Chinese University of Hong Kong, Hong Kong SAR, Hong Kong; 9 Department of Public Health, La Trobe University, Melbourne, VIC 3086 Australia

**Keywords:** Loneliness, Older adult, Meaning-centred intervention, Mixed-methods systematic review

## Abstract

**Background:**

Loneliness can pose serious health and psychological concerns among community-dwelling older adults. Meaning-centred interventions, which aim to help individuals find meaning in their lives, appeared to alleviate loneliness among older adults. Yet, systematic evidence on the effectiveness of meaning-centred intervention and the experience among older adults towards this intervention is lacking. This review will systematically synthesise the evidence to examine the effect of meaning-centred intervention on loneliness among community-dwelling older adults.

**Methods:**

A mixed-methods systematic review (MMSR) with a convergent segregated approach will be employed according to the Joanna Briggs Institute framework for MMSRs. Relevant studies will be searched from inception to 31 December 2024 from nine databases: MEDLINE (PubMed), PsycINFO, CINAHL, Scopus, Embase, Cochrane Library, ProQuest Social Science, Wangfang, and Google Scholar. Quantitative, qualitative, and mixed-methods study designs will be included. Two authors will independently perform data extraction and complete risk of bias and quality assessment using recommended tools. The evidence quality will be assessed using the Grading of Recommendations Assessment, Development and Evaluation (GRADE) approach and the Confidence in the Evidence from Reviews of Qualitative Research (CERQual) guidelines approach for quantitative and qualitative studies, respectively. The phenomena of interest will be the experience of meaning-centred intervention among older adults living in the community and long-term care facilities. The primary outcome will be loneliness. Other related outcomes include meanings in life, social connections, social participation, social isolation, suicide ideation, anxiety, and depressive symptoms.

**Discussion:**

The review will contribute to a comprehensive understanding of meaning-centred intervention on loneliness among community-dwelling older adults by integrating the quantitative and qualitative evidence. The findings will provide practitioners, researchers, and policy workers with insights on developing and/or adopting meaning-centred interventions for alleviating the loneliness of older adults in the community and eventually promoting healthy ageing.

**Systematic review registration:**

PROSPERO CRD 42024614173.

**Supplementary Information:**

The online version contains supplementary material available at 10.1186/s13643-025-02843-x.

## Background

The World Health Organization (WHO) suggested that loneliness is an important, yet neglected, social determinant of health among older adults [1]. Loneliness refers to the subjective negative experience of deficit between actual and desired levels of social connections [1, 2]. Antecedents consistently relating to loneliness among older adults include age, gender, marital status, education, income, health, number of family members in a household, living arrangements, and social support [3]. A meta-analytic review showed that loneliness increased all-cause mortality by 26% [4], which was comparable to obesity and physical inactivity [1]. A population-based cross-sectional study in Germany showed that loneliness increased the risk of depression (adjusted odds ratio: *AOR* = 1.91, 95% *CI* 1.74–2.09), anxiety (*AOR* = 1.21, 95% *CI* 1.09–1.34), and suicidal ideation (*AOR* = 1.35, 95% *CI* 1.19–1.44) [5]. A systematic review and meta-analysis involving studies from 113 countries showed that 5.25 to 24.20% of older adults suffered severe loneliness [6], which was much higher than depression (~ 7%) and anxiety (~ 3.8%) among the world’s older population [7]. As such, there is a significant need for strategies to reduce the negative effects of loneliness among older adults.

There are three types of loneliness, namely (i) social loneliness, (ii) emotional loneliness, and (iii) existential loneliness. Social loneliness refers to the perceived deficit in the quality of social connections. Emotional loneliness refers to the absence of meaningful relationships. Existential loneliness refers to a feeling of fundamental separateness from others and the wider world (e.g. lack of purpose). These subjective experiences are distinct but interrelated, influencing and exacerbating each other [8]. For example, emotional loneliness can precede the development of depressive symptoms, which in turn can lead to increased social loneliness, creating a potential vicious cycle [9]. A recent meta-synthesis on loneliness among older adults further proposed a framework of the existential human core of loneliness in old age [10]. According to the framework [10], lacking meaning in life and the accompanying feeling of emptiness are the core characteristics of loneliness in old age [10]. Another cross-sectional study showed that lack of meaning in life is a major correlate of loneliness [11]. Meaning in life is understood as an individual’s ability to attach value and significance to his or her existence [11, 12]. Antecedents of lack of meaning in life involve lacking meaningful relationships (e.g. death of a spouse) or a perceiving deficit in the quality of social connections (e.g. I am useless to others) [10, 13]. Older adults are particularly vulnerable to losing this sense of meaning due to factors such as declining health, changes in family dynamics due to the loss of a spouse or children moving away, and reduced social interaction with peers after retirement [10, 14]. Therefore, it is important to address the existential dimension (i.e. lack of meanings in life), alongside psychosocial strategies [15].

According to Gardiner, Geldenhuys, and Gott [16], there are six categories of interventions for loneliness, namely: (i) social facilitation interventions, (ii) psychological therapies, (iii) health and social care provision, (iv) animal interventions, (v) befriending interventions, and (vi) leisure/skill development. An umbrella review [17] was conducted to review 16 systematic reviews of effectiveness of interventions addressing loneliness in older adults for the period 2000–2017. Jarvis et al. [17] extracted data from 14 randomised controlled trials and found that interventions on loneliness were limited, with effect sizes ranging from − 1.23 to 0.44, with the greatest effect size in a social cognition intervention that aimed to correct the maladaptive etiquette during social interaction. There was inconclusive evidence on the effectiveness of interventions to alleviate loneliness among older adults, particularly for the existential dimension.

While most interventions for loneliness aimed to improve social connections [16, 17], they paid little attention to address the issue of lack of meaning in life [11]. Based on the framework of the existential human core of loneliness in old age, meaning-centred intervention can be an effective intervention for alleviating loneliness among older adults. An example of meaning-centred intervention was the meaning-centred group psychotherapy grounded in Viktor Frankl’s writings to help patients with advanced cancer sustain or enhance a sense of meaning, peace, and purpose in their lives [18]. Vitkor Frankl suggested that (i) there are always meanings in life, even in the most suffering situation, (ii) humans are motivated to search for meanings, and (iii) humans are free to search for meanings [19]. Viktor Frankl suggested that modern Western society tends to adore the young and achievement orientation, which focuses on present usefulness, and consequently downplayed those who had previously contributed to society but may now have become less capable of productivity according to the modern world standard due to functional decline and chronic illnesses. This creates a lack of meaning in older life [19]. Therefore, restoring meaning in older adults is essential. There are emerging evidences showing the effectiveness of meaning-centred intervention to alleviate loneliness and restore meaning in life among people at the end-of-life stages [18, 20] and older adults [21]. In addition to restoring meanings in life, meaning-centred intervention, particularly in group, was also suggested to promote interpersonal connectedness by encouraging communication of one’s experiences and meanings within a group. This open communication may help facilitate group support and a sense of belonging and, therefore, may further reduce social and emotional loneliness [20]. Therefore, meaning-centred interventions may provide an effective foundation to enhance current strategies, as discussed by Gardiner et al. [16].

Apart from examining the effectiveness, it may be equally important to examine the experience of older adults with loneliness who received meaning-centred intervention. These findings may help to reveal the mechanism—how the meaning-centred intervention may help reduce loneliness, and what older adults with loneliness appreciate or would like to improve in the intervention. However, a systematic review on the effectiveness of meaning-centred interventions on loneliness among older adults, and the experiences of older adults with loneliness receiving meaning-centred intervention, was lacking. Understanding both the effectiveness of loneliness and experiences of meaning-centred intervention among community-dwelling older adults will provide a comprehensive picture of how to develop future successful practices.

## Methods

This is a mixed-methods systematic review (MMSR) that will employ a convergent segregated approach and will be conducted by following the Joanna Briggs Institute (JBI) framework for MMSRs [22]. This review protocol was prepared using the Preferred Reporting Items for Systematic reviews and Meta-Analyses Protocol (PRISMA-P) guidelines [23]. The PRISMA-P checklist was completed (Additional file 1). This protocol was prospectively registered in PROSPERO on 28 November 2024 (CRD 42024614173).

### Objectives

This MMSR will be conducted to synthesise the current evidence regarding the meaning-centred intervention for managing loneliness among community-dwelling older adults. The research questions are as follows:

1. What are the effects of meaning-centred interventions on the loneliness and other related outcomes (e.g. meanings in life and social connection) of community-dwelling older adults?
2. What is the experience of older adults during or after meaning-centred intervention?

### Eligibility criteria

The quantitative and qualitative studies or components will be included according to the inclusion and exclusion criteria based on the population, intervention, comparison and outcome (PICO) and population, interest and context (PICo) frameworks, respectively [24] (see Table 1). The population will be the older adults who are aged 60 years and over and live in the community and the long-term care facilities. The intervention will be a meaning-centred intervention, which is defined as an intervention that aims to help individuals find meaning in their lives [25]. Examples include but are not limited to logotherapy and existential therapy [26]. The phenomena of interest will be the experience of meaning-centred intervention among older adults living in the community and long-term care facilities. The primary outcome of this review is loneliness. Other related outcomes include meanings in life, social connections, social participation, social isolation, suicide ideation, anxiety, and depressive symptoms. Studies on other outcomes not mentioned above will be considered to give comprehensive insight into the impact of meaning-centred intervention on older adults. However, studies that do not evaluate the primary outcome will be excluded. All outcome measures have to be assessed at baseline and after the interventions. The context of the review will be the community setting, including long-term care facilities.

**Table 1:** Eligibility criteria based on the PICO and PICo framework

	Quantitative studies	Qualitative studies
Population (P)/participants (P)	Older adult, aged 60 years and over, and live in the community- or long-term care facilities	
Intervention (I)/phenomenon of interest (I)	Meaning-centred intervention is defined as an intervention that aims to help individuals find meaning in their lives (1). Examples include but are not limited to logotherapy and existential therapy	Experience of meaning-centred intervention
Comparator (C)/context (Co)	The quantitative component of the review considers studies in which the comparison group received either an alternative intervention (e.g. acceptance and commitment therapy), no intervention, or waitlist control	Community setting and long-term care facilities
Outcome (O)	• The primary outcome of this review is loneliness • Loneliness in this review is defined as a psychological state of distress or discomfort that results when one perceives a gap between one’s desires for social connection and actual experiences of it • The other related outcomes include meaning in life, social participation, social isolation, social connections, suicide ideation, anxiety and depressive symptoms, social well-being). Studies on other outcomes not mentioned above are considered to give comprehensive insight into the impact of meaning-centred intervention on older adults. However, studies that do not evaluate the primary outcome are excluded. All outcome measures have to be evaluated at baseline and after the interventions	

This review will consider randomised controlled trials (RCTs), quasi-experimental studies, qualitative studies, and mixed-methods studies. Mixed-methods studies will be considered if the data from the quantitative or qualitative components can be extracted separately. If the quantitative and qualitative data cannot be separated, the study will be used for qualitative synthesis. Studies published in English and Chinese will be included. Case reports, conference abstracts, posters, comments, and dissertations will be excluded.

### Search strategy

The search strategy will aim to locate both published and unpublished studies. A three-step search strategy will be utilised in this review. First, an initial limited search of MEDLINE (PubMed), PsycINFO, ProQuest Social Science, Wangfang, and Google Scholar will be undertaken to identify related articles. The text words in the titles and abstracts of relevant articles and the index terms will be used to develop a full search strategy. Subsequently, MEDLINE (PubMed), PsycINFO, Cumulative Index to Nursing and Allied Health Literature (CINAHL), Scopus, Embase, Cochrane Library, ProQuest Social Science, Wanfang, and Google Scholar will be searched from inception until 31 December 2024. These databases provide high-quality and vast references from peer-reviewed journals, covering broad healthcare disciplines such as medicine, nursing, allied health professionals, psychology, and social science. In particular, Wanfang covers references published in Chinese, and Google Scholar covers grey literature. Thus, coverage of the literature included in this MMSR is comprehensive. Reference lists of the included studies, related systematic reviews, and meta-analyses will be searched to identify additional relevant studies. The keywords will be around the PICO and PICo frameworks, i.e. older adults, meaning-centred intervention, loneliness, and community-dwelling. Table 2 presents the related search terms.

**Table 2:** The search terms

Population	Older adults, older people, elderly, aged (MeSH), geriatric, older adulthood, elder care	
Intervention	Logotherapy (MeSH), meaning centred, meaning based, meaning focused, logotherap*, existential therapy, spiritual intervention, spiritual	
Comparator/context	Active control, passive control, waitlist control	Community setting, long-term care facilities, residential
Outcome	Loneliness (MeSH), isolation, lone*	Experience

### Study selection

All identified citations will be collated and imported into EndNote (The EndNote Team 2020. EndNote 20 ed, Philadelphia, PA, USA: Clarivate), and duplicates will be removed. If abstracts or articles are not reported in English, they will have to be translated into English using Google Translate (https://translate.google.com). The first 10 studies at each screening step will be pilot-screened to ensure consistency between reviewers. Titles and abstracts will then be screened by two independent reviewers (K. H. and J. C.) for assessment against the inclusion criteria for the review. Potentially relevant studies will be retrieved in full, and their citation details will be checked for additional relevant studies. The full text of selected studies will be assessed in detail against the inclusion criteria by two independent reviewers. Reasons for exclusion of full-text studies that do not meet the inclusion criteria will be recorded and reported in the systematic review. Any disagreements that arise between the reviewers at each stage of the study selection process will be resolved through discussion or with a third reviewer (D. C.). The search results will be reported in full in the final report and presented in a PRISMA flow diagram [27].

### Quality appraisal

Quantitative studies (and a quantitative component of mixed-methods studies) selected for retrieval will be assessed by two independent reviewers (K. H. and J. C.) for methodological validity prior to inclusion in the review using the JBI standardised critical appraisal instruments for quasi-experimental studies [28] and randomised controlled trials [29]. Qualitative studies (and qualitative component of mixed methods studies) selected for retrieval will be assessed by two independent reviewers for methodological validity prior to inclusion in the review using the JBI standardised critical appraisal instrument for qualitative research [24].

Where required, paper authors will be contacted to request missing or additional data for clarification. Any disagreements that arise between the reviewers will be resolved through discussion. All studies will be considered to be of adequate quality for data extraction and synthesis if they achieve at least 50% of the total score of the critical appraisal instrument. Critical appraisal results will be reported in narrative form and a table, and they will be included in the discussion of data synthesis.

### Data extraction

A standardised extraction form will be developed and tested prior to data extraction. Two reviewers (K. H. and J. C.) will independently chart the data, and any discrepancies will be resolved by discussion and consensus. The characteristics of included studies to be extracted will be included, but not limited to author(s), publication year, country, study aim, design, settings, sampling and sample size, characteristics of participants (mean age, sex, and number of participants), and characteristics of interventions (aim, underpinning theory, intervention components, duration, and frequency). After that, quantitative outcomes representing the effects (tools used to measure loneliness and other related outcomes and study findings, i.e. change in loneliness and other related outcomes) will be extracted from the quantitative studies and the quantitative component of any mixed-methods studies.

The extraction of data from qualitative studies and the qualitative component of mixed-methods studies will be conducted by the two independent reviewers (K. H. and J..C). The findings (e.g. a verbatim extract of the included study, the author’s analytical interpretation of the results or data) and illustrations (e.g. a direct quote) of the older adults’ experiences of the meaning-centred intervention will be extracted. The two independent reviewers will discuss and allocate three levels of plausibility to the extracted data, namely: (i) unequivocal (findings accompanied by an illustration that is beyond reasonable doubt and therefore not open to challenge), (ii) equivocal (findings accompanied by an illustration lacking clear association with it and therefore open to challenge), and (iii) unsupported (findings are not supported by the data). Any disagreements that arise between the reviewers in the data extraction process will be resolved through discussion or with an additional reviewer (D. C.). Study authors will be contacted for any missing or unreported data at a maximum of three e-mails. If data remains unavailable, analysis will be done only on the available data, and the potential impact of missing data will be reported in the “ Discussion” section.

### Data synthesis and integration

A convergent segregated approach will be used to synthesise and integrate the data, in accordance with the JBI methodology for MMSRs [22], which addresses questions about the effectiveness of the intervention and the experience. This will involve separate quantitative and qualitative synthesis followed by integration of the resultant quantitative and qualitative evidence.

### Quantitative synthesis

Meta-analysis will be performed to evaluate the effectiveness of the intervention by using Review Manager 5.4 only on RCTs. Outcomes that are measured as continuous data and by different scales among RCTs will be extracted and converted to standardised mean differences (SMDs). Should the RCTs have more than two intervention groups, a single pair-wise comparison will be created by combining groups according to the guideline from the Cochrane Handbook [30], such that a meaning-centred intervention group will compare with a group of usual treatment (e.g. waitlist control, reminiscence therapy). The SMDs will be pooled to estimate the effect size, which will be presented as Cohen’s d with 95% confidence intervals and a significant level at 0.05. An effect size of less than 0.2, 0.5, and 0.8 will be considered to indicate a small, moderate, and large effect, respectively. The heterogeneity among studies will be measured by conducting *I*^2^ tests. High values (*I*^2^ > 50%) indicate the presence of heterogeneity, and a random effects model will be used. Otherwise, fixed-effect model will be used.

To help address the heterogeneity of the included studies, sub-group analyses will be performed based on demographic characteristics (e.g. community vs long-term care facilities) in case of significant heterogeneity. A funnel plot will be generated by using Review Manager 5.4 to assess publication bias if there are 10 or more studies included in a meta-analysis. Statistical tests for funnel plot asymmetry (Egger test, Begg test, Harbord test) will be performed where appropriate. Where statistical pooling is impossible, the findings will be presented in narrative form, including tables and figures where applicable to aid in data presentation.

### Qualitative synthesis

The qualitative data related to the intervention’s experience will be pooled using the meta-aggregation approach [24]. Only unequivocal and equivocal findings will be included in the meta-aggregation. This will involve aggregating findings to generate a set of statements that represent that aggregation, assembling the findings and categorizing them based on similarity in meaning. These categories will then be subjected to a synthesis to produce a comprehensive set of synthesised findings.

### Integration of quantitative evidence and qualitative evidence

The synthesised quantitative and qualitative findings will be integrated using a configurative analysis. The reviewers (K. H. and J. C.) will repeatedly juxtapose the results of the synthesised quantitative evidence with the synthesised qualitative findings, analysing the meaning-centred intervention that had been investigated for effectiveness on loneliness in light of the experiences of the older adults. Five questions recommended by JBI [22] will be used to organise or link the evidence from both syntheses into a line of argument. They are as follows:

(i) Are the results/findings from individual synthesis supportive or contradictory?
(ii) Does the qualitative evidence explain why the intervention is or is not effective?
(iii) Does the qualitative evidence help explain differences in the direction and size of effect across the included quantitative studies?
(iv) Which aspects of the quantitative evidence are/are not explored in the qualitative studies?
(v) Which aspects of the qualitative evidence are/are not tested in the quantitative evidence?

The integrated evidence will be presented in the form of configuration. Where configuration is not possible, the findings will be presented in a narrative format.

### Accessing confidence in evidence

For the quantitative evidence, the Grading of Recommendations Assessment, Development and Evaluation (GRADE) approach [31] will be used to assess the confidence in the evidence of effectiveness arising from studies evaluating the effectiveness of meaning-centred intervention. We will present our GRADE assessments in a summary of findings table. For the qualitative evidence, the Confidence in the Evidence from Reviews of Qualitative Research (CERQual) [32] approach will be used to assess confidence in the qualitative evidence synthesis regarding the experience of older adults during or after meaning-centred intervention. The CERQual findings will be summarised and presented in a summary of qualitative findings table.

## Discussion

Loneliness is an important, yet neglected, social determinant of health among older adults, amid the ageing population worldwide. The increased risk of developing various psychological conditions, including depression, anxiety, and suicidal ideation, associated with loneliness is anticipated [5]. Therefore, it is imperative to provide effective interventions to alleviate loneliness for this vulnerable population. Emerging evidence shows that meaning-centred intervention might alleviate loneliness by restoring meaning in life among older adults [21]. However, there is a lack of systematic review on the effectiveness of meaning-centred interventions on loneliness among older adults and the experiences of older adults with loneliness receiving meaning-centred intervention. This review will help fill this gap by systematically synthesising the evidence to examine the effect of loneliness and explore the experience of meaning-centred intervention among community-dwelling older adults.

A potential limitation of this proposal may arise from the extensiveness of the search for potential articles. To address this potential challenge, articles published in English and Chinese will be included. Additionally, an exhaustive search strategy will be adopted, including identifying the key terms from four relevant databases, searching the potential articles with the identified key terms from eight relevant databases, and identifying additional articles by manually searching the included articles’ reference list.

Despite the limitations, the proposed study has several strengths. To our knowledge, this will be the first MMSR to systematically appraise the quantitative and qualitative evidence on meaning-centred intervention among community-dwelling older adults. This MMSR will provide a comprehensive understanding of the effectiveness and mechanism of the meaning-centred intervention on loneliness among older adults in the community. Subsequently, the findings will provide practitioners, researchers, and policy workers with insights on developing and/or adopting meaning-centred interventions for alleviating the loneliness of older adults in the community and eventually promoting healthy aging.

## Supplementary Information

Additional file 1. PRISMA-P 2015 Checklist

## Data Availability

The datasets used and/or analysed during the current study are available from the corresponding author on reasonable request.

## Abbreviations

AOR: Adjusted odds ratio
CERQual: Confidence in the Evidence from Reviews of Qualitative Research
CI: Confidence interval
GRADE: Grading of Recommendations Assessment, Development and Evaluation
JBI: Joanna Briggs Institute
MMSR: Mixed-methods systematic review
PRISMA: Preferred Reporting Items for Systematic reviews and Meta-Analyses
PROSPERO: International Prospective Register of Systematic Reviews
RCT: Randomised controlled trial
SMD: Standardised mean difference
WHO: World Health Organization
